# Supplementary material for: Hybrid Models and Biological Model Reduction with PyDSTool
Source: PLoS Comput Biol. 2012 Aug 9;8(8):e1002628. doi: 10.1371/journal.pcbi.1002628 (PMC3415397; doi:10.1371/journal.pcbi.1002628)
Supplement: Text S4 — Complete source code for the PyDSTool package (version 0.88.120504). Includes API documentation and help files linking to web pages. This file is identical to the current public release on Sourceforge.net. (ZIP) [file pcbi.1002628.s004.zip › PyDSTool/html/PyDSTool.Model.Model-class.html]

xml version="1.0" encoding="ascii"?


PyDSTool.Model.Model


| Home | Trees | Indices | Help | PyDSTool |
| --- | --- | --- | --- | --- |

Package PyDSTool ::
Module Model ::
Class Model

[hide private]

[frames] | no frames]

# Class Model

source code

```
object --+
         |
        Model
```

Known Subclasses:
:   - HybridModel
    - , NonHybridModel

---

General-purpose Hybrid and Non-Hybrid Model abstract class.


|  |  |
| --- | --- |
| Instance Methods | [hide private] |

|  |  |
| --- | --- |
| \_\_init\_\_(self, legit, \*a, \*\*kw)  x.\_\_init\_\_(...) initializes x; see x.\_\_class\_\_.\_\_doc\_\_ for signature | source code |

|  |  |
| --- | --- |
| \_\_len\_\_(self)  Return number of sub-models | source code |

|  |  |
| --- | --- |
| sub\_models(self)  Return a list of all sub-model instances (model interfaces or generators) | source code |

|  |  |
| --- | --- |
| \_makeDefaultVarNames(self)  Return default observable, internal, and auxiliary variable names from modelInfo. | source code |

|  |  |
| --- | --- |
| \_generateParamInfo(self)  Record parameter info locally, for future queries. | source code |

|  |  |
| --- | --- |
| showDef(self, target=None, type=`'``'`)  type = 'spec', 'auxspec', 'auxfnspec', 'events', or 'modelspec' (leave blank for the first \*four\* together). | source code |

|  |  |
| --- | --- |
| showSpec(self) | source code |

|  |  |
| --- | --- |
| showAuxSpec(self) | source code |

|  |  |
| --- | --- |
| showAuxFnSpec(self) | source code |

|  |  |
| --- | --- |
| showEventSpec(self) | source code |

|  |  |
| --- | --- |
| current\_defining\_args(self) | source code |

|  |  |
| --- | --- |
| has\_exact\_traj(self, trajname, info)  Compare self.pars, self.icdict and self.tdata against what's stored for a previously computed trajectory, so that re-computation can be avoided. | source code |

|  |  |
| --- | --- |
| \_prepareCompute(self, trajname, \*\*kw) | source code |

|  |  |
| --- | --- |
| query(self, querykey=`'``'`)  Return info about Model set-up. | source code |

|  |  |
| --- | --- |
| getEventMappings(self, dsName) | source code |

|  |  |
| --- | --- |
| setPars(self, p, val) | source code |

|  |  |
| --- | --- |
| setICs(self, p, val) | source code |

|  |  |
| --- | --- |
| set(self, \*\*kw)  Set specific parameters of Model. | source code |

|  |  |
| --- | --- |
| \_\_getitem\_\_(self, trajname) | source code |

|  |  |
| --- | --- |
| \_\_delitem\_\_(self, trajname) | source code |

|  |  |
| --- | --- |
| \_delTraj(self, trajname)  Delete a named trajectory from the database. | source code |

|  |  |
| --- | --- |
| \_\_call\_\_(self, trajname, t, coords=None, asGlobalTime=True, asmap=False)  Evaluate position of hybrid trajectory at time t. | source code |

|  |  |
| --- | --- |
| getEndPoint(self, trajname, end=1)  Returns endpoint of specified trajectory as Point. | source code |

|  |  |
| --- | --- |
| getEndTime(self, trajname, end=1)  Returns end time of specified trajectory. | source code |

|  |  |
| --- | --- |
| \_validateVarNames(self, names)  Check types and uniqueness of variable names. | source code |

|  |  |
| --- | --- |
| forceObsVars(self, varnames)  Force variables to be the observables in the Model. | source code |

|  |  |
| --- | --- |
| forceIntVars(self, varnames)  Force variables to become internal variables in the Model. | source code |

|  |  |
| --- | --- |
| defaultVars(self)  (Re)set to default observable and internal variable names. | source code |

|  |  |
| --- | --- |
| info(self, verboselevel=1) | source code |

|  |  |
| --- | --- |
| \_\_repr\_\_(self)  str(x) | source code |

|  |  |
| --- | --- |
| \_\_str\_\_(self)  str(x) | source code |

|  |  |
| --- | --- |
| \_\_copy\_\_(self) | source code |

|  |  |
| --- | --- |
| \_\_deepcopy\_\_(self, memo=None, \_nil=`[``]`) | source code |

|  |  |
| --- | --- |
| renameTraj(self, trajname, newname, force=False)  Rename stored trajectory. | source code |

|  |  |
| --- | --- |
| getTrajModelName(self, trajname, t=None)  Return the named trajectory's associated sub-model(s) used to create it, specific to time t if given. | source code |

|  |  |
| --- | --- |
| getTrajEventTimes(self, trajname, events=None)  Return the named trajectory's Generator-flagged event times. | source code |

|  |  |
| --- | --- |
| getTrajEvents(self, trajname, events=None)  Return the named trajectory's Generator-flagged events. | source code |

|  |  |
| --- | --- |
| getTrajEventStruct(self, trajname)  Return the named trajectory's model event structure (representing external constraints), as present when it was computed. | source code |

|  |  |
| --- | --- |
| getTrajTimeInterval(self, trajname)  Return the named trajectory's time domain, over which it is defined, in a single interval. | source code |

|  |  |
| --- | --- |
| getTrajTimePartitions(self, trajname)  Return the named trajectory's time domain, over which it is defined, as a list of time interval partitions. | source code |

|  |  |
| --- | --- |
| getDSAlgPars(self, target, par, idx=None)  Returns value of given algorithmic parameter for selected sub-model. | source code |

|  |  |
| --- | --- |
| setDSAlgPars(self, target, par, val)  Set value of algorithmic parameter in a specific generator. | source code |

|  |  |
| --- | --- |
| sample(self, trajname, coords=None, dt=None, tlo=None, thi=None, doEvents=True, precise=False)  Uniformly sample the named trajectory over range indicated, including any event points. | source code |

|  |  |
| --- | --- |
| \_infostr(self, dsName=None, verbosity=0)  Return string information about named sub-model (if given by dsName) at given verbosity level (default 0) | source code |

|  |  |
| --- | --- |
| showDSEventInfo(self, target, verbosity=1, ics=None, t=0) | source code |

|  |  |
| --- | --- |
| getDSEventTerm(self, target, flagVal=True, ics=None, t=0)  List of events in target which are terminal/non-terminal according to value of flagVal. | source code |

|  |  |
| --- | --- |
| setDSEventTerm(self, target, eventTarget, flagVal, ics=None, t=0)  Set event in a specific generator to be (non)terminal. | source code |

|  |  |
| --- | --- |
| getDSEventActive(self, target, flagVal=True, ics=None, t=0)  List of events in target which are active/inactive according to value of flagVal. | source code |

|  |  |
| --- | --- |
| setDSEventActive(self, target, eventTarget, flagVal, ics=None, t=0)  Set event in a specific generator to be (in)active. | source code |

|  |  |
| --- | --- |
| setDSEventICs(self, target, eventTarget, val, ics=None, t=0)  Set event in a specific generator to have specified initial conditions. | source code |

|  |  |
| --- | --- |
| setDSEventPrecise(self, target, eventTarget, flagVal, ics=None, t=0)  Set event in a specific generator to be (im)precise. | source code |

|  |  |
| --- | --- |
| setDSEventDelay(self, target, eventTarget, val, ics=None, t=0)  Set event in a specific generator to have specified event delay. | source code |

|  |  |
| --- | --- |
| setDSEventInterval(self, target, eventTarget, val, ics=None, t=0)  Set event in a specific generator to have specified event interval. | source code |

|  |  |
| --- | --- |
| setDSEventTol(self, target, eventTarget, val, ics=None, t=0)  Set event in a specific generator to have specified event tolerance. | source code |

|  |  |
| --- | --- |
| setDSEventDir(self, target, eventTarget, val, ics=None, t=0)  Set event in a specific generator to have specified direction code. | source code |

|  |  |
| --- | --- |
| setDSEventStartTime(self, target, eventTarget, val, ics=None, t=0)  Set event in a specific generator to have specified start time. | source code |

|  |  |
| --- | --- |
| setDSEventBisect(self, target, eventTarget, val, ics=None, t=0)  Set event in a specific generator to have specified bisect limit. | source code |

|  |  |
| --- | --- |
| resetEventTimes(self)  Internal method | source code |

|  |  |
| --- | --- |
| \_set\_for\_hybrid\_DS(self, state)  Internal method to set all sub-models flag for being part of a hybrid trajectory computation. | source code |

|  |  |
| --- | --- |
| searchForNames(self, template)  Find parameter / variables names matching template in the model's generators or sub-models: the returned result is a list. | source code |

|  |  |
| --- | --- |
| searchForVars(self, template)  Find variable and auxiliary variable names that have to be in every generator or sub-model: the returned result is a list. | source code |

**Inherited from `object`**:
`__delattr__`,
`__getattribute__`,
`__hash__`,
`__new__`,
`__reduce__`,
`__reduce_ex__`,
`__setattr__`


|  |  |
| --- | --- |
| Class Variables | [hide private] |

\_needKeys = `['name', 'modelInfo']`

\_optionalKeys = `['ics', 'mspecdict', 'verboselevel', 'norm', '...`

\_querykeys = `['pars', 'parameters', 'events', 'submodels', 'ic...`

\_setkeys = `['pars', 'algparams', 'checklevel', 'abseps', 'ics'...`


|  |  |
| --- | --- |
| Properties | [hide private] |

**Inherited from `object`**:
`__class__`


|  |  |
| --- | --- |
| Method Details | [hide private] |

|  |  |
| --- | --- |
| \_\_init\_\_(self, legit, \*a, \*\*kw)  *(Constructor)* | source code |

x.\_\_init\_\_(...) initializes x; see x.\_\_class\_\_.\_\_doc\_\_ for
signature

Overrides: object.\_\_init\_\_: *(inherited documentation)*

|  |  |
| --- | --- |
| \_generateParamInfo(self) | source code |

Record parameter info locally, for future queries. Internal use
only.

|  |  |
| --- | --- |
| showDef(self, target=None, type=`'``'`) | source code |

type = 'spec', 'auxspec', 'auxfnspec', 'events', or 'modelspec' (leave
blank for the first \*four\* together).

'spec', 'auxspec' and 'auxfnspec', 'events' refer to the compiled
target language code for the specifications. 'modelspec' refers to the
pre-compiled abstract specifications of the model.

|  |  |
| --- | --- |
| query(self, querykey=`'``'`) | source code |

```
Return info about Model set-up.
Valid query key: 'pars', 'parameters', 'events', 'submodels',
 'ics', 'initialconditions', 'vars', 'variables',
 'auxvars', 'auxvariables', 'vardomains', 'pardomains', 'abseps'
```

|  |  |
| --- | --- |
| set(self, \*\*kw) | source code |

```
Set specific parameters of Model. These will get passed on to
all Generators/sub-models that support these keys unless the
restrictDSList argument is set (only applies to keys algparams,
checklevel, and abseps).

Permitted keys: 'pars', 'algparams', 'checklevel', 'abseps',
                'ics', 'inputs', 'tdata', 'restrictDSlist',
                'globalt0', 'verboselevel', 'inputs_t0'
```

|  |  |
| --- | --- |
| \_\_call\_\_(self, trajname, t, coords=None, asGlobalTime=True, asmap=False)  *(Call operator)* | source code |

Evaluate position of hybrid trajectory at time t. if optional argument
asmap == True then t must be an integer in [0, #segments].

|  |  |
| --- | --- |
| getEndPoint(self, trajname, end=1) | source code |

Returns endpoint of specified trajectory as Point. trajname: name of
selected trajectory end: (default=1) index of trajectory endpoint. 0
=> first, 1 => last

|  |  |
| --- | --- |
| getEndTime(self, trajname, end=1) | source code |

Returns end time of specified trajectory. trajname: name of selected
trajectory end: (default=1) index of trajectory endpoint. 0 => first,
1 => last

|  |  |
| --- | --- |
| forceObsVars(self, varnames) | source code |

Force variables to be the observables in the Model. May also promote
auxiliary variables.

|  |  |
| --- | --- |
| forceIntVars(self, varnames) | source code |

Force variables to become internal variables in the Model. May also
promote auxiliary variables.

|  |  |
| --- | --- |
| \_\_repr\_\_(self)  *(Representation operator)* | source code |

str(x)

Overrides: object.\_\_repr\_\_: *(inherited documentation)*

|  |  |
| --- | --- |
| \_\_str\_\_(self)  *(Informal representation operator)* | source code |

str(x)

Overrides: object.\_\_str\_\_: *(inherited documentation)*

|  |  |
| --- | --- |
| renameTraj(self, trajname, newname, force=False) | source code |

Rename stored trajectory. Force option (default False) will overwrite
any existing trajectory with the new name.

|  |  |
| --- | --- |
| getTrajEventTimes(self, trajname, events=None) | source code |

Return the named trajectory's Generator-flagged event times.

events argument can be singleton string name of an event, returning
the event data, or events can be a list of event names, returning a
dictionary of event name -> event data. Event names should use
hierarchical naming convention, if applicable.

|  |  |
| --- | --- |
| getTrajEvents(self, trajname, events=None) | source code |

Return the named trajectory's Generator-flagged events.

events argument can be singleton string name of an event, returning
the event data, or events can be a list of event names, returning a
dictionary of event name -> event data. Event names should use
hierarchical naming convention, if applicable.

|  |  |
| --- | --- |
| getDSAlgPars(self, target, par, idx=None) | source code |

```
Returns value of given algorithmic parameter for selected sub-model.
target -- name of sub-model in model (cannot be list).
par -- name of algorithmic parameter.
idx -- (optional) index into value if algorithmic parameter val is a
  list of values.
```

|  |  |
| --- | --- |
| setDSAlgPars(self, target, par, val) | source code |

```
Set value of algorithmic parameter in a specific generator.
target -- name or list of generators in model.
par -- name of algorithmic parameter is to be set.
val -- value to which the algorithmic parameter is to be set.

if target is a list, then algorithmic pararameter 'par' is
set to 'val' for every generator in the list, if par exists for that
generator.

WARNING: THIS FUNCTION IS NOT 'SAFE' -- IT DOES NOT CHECK THAT VALS
  ARE APPROPRIATE TO PARAMETERS!!!
```

|  |  |
| --- | --- |
| sample(self, trajname, coords=None, dt=None, tlo=None, thi=None, doEvents=True, precise=False) | source code |

```
Uniformly sample the named trajectory over range indicated,
including any event points. (e.g. use this call for plotting purposes.)
Outputs a Pointset from the trajectory over a given range.

Arguments:

  trajname   Name of stored trajectory to sample
  coords     (optional) list of variable names to include in output
  dt         (optional) step size to use in sampling of independent variable
             If not given, the underlying time mesh is used, if available.
  tlo        (optional) Start value for independent variable, default 0
  thi        (optional) End value for independent variable, default last value
  doEvents   (optional) include any event points in output, default True
  precise    (optional) The default value, False, causes an attempt to use
             the underlying mesh of the trajectory to return a Pointset more
             quickly. Currently, this can only be used for trajectories that
             have a single segment (non-hybrid).
```

|  |  |
| --- | --- |
| getDSEventTerm(self, target, flagVal=True, ics=None, t=0) | source code |

List of events in target which are terminal/non-terminal according to
value of flagVal. target -- name of generators in model (cannot be list)
flagVal -- True (terminal) or False (non-terminal)

|  |  |
| --- | --- |
| setDSEventTerm(self, target, eventTarget, flagVal, ics=None, t=0) | source code |

```
Set event in a specific generator to be (non)terminal.
target -- name or list of names of generators in model.
eventTarget -- name or list of names of events in specified generator(s)
flagVal -- True (event terminal) or False (event non-terminal)

flagVal is applied to every listed event in every listed generator, if
  events and generators exist.
```

|  |  |
| --- | --- |
| getDSEventActive(self, target, flagVal=True, ics=None, t=0) | source code |

List of events in target which are active/inactive according to value
of flagVal. target -- name of generators in model (cannot be list)
flagVal -- True (active) or False (inactive)

|  |  |
| --- | --- |
| setDSEventActive(self, target, eventTarget, flagVal, ics=None, t=0) | source code |

Set event in a specific generator to be (in)active. target -- name or
list of names of generators in model. eventTarget -- name or list of
names of events in specified generator(s) flagVal -- True (event active)
or False (event inactive)

flagVal is applied to every listed event in every listed generator, if
events and generators exist.

|  |  |
| --- | --- |
| setDSEventICs(self, target, eventTarget, val, ics=None, t=0) | source code |

Set event in a specific generator to have specified initial
conditions. target -- name or list of names of generators in model.
eventTarget -- name or list of names of events in specified generator(s)
val -- dictionary of varnames and initial condition values

val is applied to every listed event in every listed generator, if
events and generators exist. If a varname in the val dict does not exist
in a specified generator/event, then it is skipped.

|  |  |
| --- | --- |
| setDSEventPrecise(self, target, eventTarget, flagVal, ics=None, t=0) | source code |

Set event in a specific generator to be (im)precise. target -- name or
list of names of generators in model. eventTarget -- name or list of
names of events in specified generator(s) flagVal -- True (event precise)
or False (event imprecise)

flagVal is applied to every listed event in every listed generator, if
events and generators exist.

|  |  |
| --- | --- |
| setDSEventDelay(self, target, eventTarget, val, ics=None, t=0) | source code |

Set event in a specific generator to have specified event delay.
target -- name or list of names of generators in model. eventTarget --
name or list of names of events in specified generator(s) val -- event
delay (int, float >= 0)

val is applied to every listed event in every listed generator, if
events and generators exist.

|  |  |
| --- | --- |
| setDSEventInterval(self, target, eventTarget, val, ics=None, t=0) | source code |

Set event in a specific generator to have specified event interval.
target -- name or list of names of generators in model. eventTarget --
name or list of names of events in specified generator(s) val -- event
interval (int, float >= 0)

val is applied to every listed event in every listed generator, if
events and generators exist.

|  |  |
| --- | --- |
| setDSEventTol(self, target, eventTarget, val, ics=None, t=0) | source code |

Set event in a specific generator to have specified event tolerance.
target -- name or list of names of generators in model. eventTarget --
name or list of names of events in specified generator(s) val -- event
tolerance (int, float >= 0)

val is applied to every listed event in every listed generator, if
events and generators exist.

|  |  |
| --- | --- |
| setDSEventDir(self, target, eventTarget, val, ics=None, t=0) | source code |

Set event in a specific generator to have specified direction code.
target -- name or list of names of generators in model. eventTarget --
name or list of names of events in specified generator(s) val --
direction code (-1: decreasing, 1: increasing, 0: either direction)

val is applied to every listed event in every listed generator, if
events and generators exist.

|  |  |
| --- | --- |
| setDSEventStartTime(self, target, eventTarget, val, ics=None, t=0) | source code |

Set event in a specific generator to have specified start time. target
-- name or list of names of generators in model. eventTarget -- name or
list of names of events in specified generator(s) val -- start time
(float, int)

val is applied to every listed event in every listed generator, if
events and generators exist.

|  |  |
| --- | --- |
| setDSEventBisect(self, target, eventTarget, val, ics=None, t=0) | source code |

Set event in a specific generator to have specified bisect limit.
target -- name or list of names of generators in model. eventTarget --
name or list of names of events in specified generator(s) val -- bisect
limit (int > 0)

val is applied to every listed event in every listed generator, if
events and generators exist.

|  |  |
| --- | --- |
| \_set\_for\_hybrid\_DS(self, state) | source code |

Internal method to set all sub-models flag for being part of a hybrid
trajectory computation. Useful to indicate that high level events should
not be reset when a Generator is reused between hybrid trajectory
segments.

  


|  |  |
| --- | --- |
| Class Variable Details | [hide private] |

### \_optionalKeys

Value:
:   ```
    ['ics',
     'mspecdict',
     'verboselevel',
     'norm',
     'tdata',
     'eventPars',
     'abseps']
    ```

### \_querykeys

Value:
:   ```
    ['pars',
     'parameters',
     'events',
     'submodels',
     'ics',
     'initialconditions',
     'vars',
     'variables',
    ...
    ```

### \_setkeys

Value:
:   ```
    ['pars',
     'algparams',
     'checklevel',
     'abseps',
     'ics',
     'inputs',
     'tdata',
     'restrictDSlist',
    ...
    ```

  


| Home | Trees | Indices | Help | PyDSTool |
| --- | --- | --- | --- | --- |

|  |  |
| --- | --- |
| Generated by Epydoc 3.0.1 on Fri May 4 15:24:07 2012 | http://epydoc.sourceforge.net |
